# American Medical Association

**Published:** 1877-08

**Authors:** 


### American Medical Association.

TWENTY-EIGHTH ANNUAL MEETING.—FIRST DAY'S PROCEEDINGS.

The Twenty-eighth Annual Meeting of the American Medical Association was held in Farwell Hall, Chicago, beginning on Tuesday June 5th. It was opened by an address from Dr. J. Marion Sims, who then presented the new president Dr. Bowdich, of Boston. Dr. N. S. Davis, of Chicago, delivered the address of welcome to Chicago. In the course of his remarks he very appropriately referred to the early organization of the association. This he was quite competent to do, for, as all our readers are aware, he was himself the leader of the effort. The association, whose annual attendance now numbers over six hundred, has shown its appreciation of his labors by twice electing him to the office of president—an honor which has been conferred upon no other physician of this country.

The sections and the association proper all immediately settled down to work, for on the first day of the session the following papers were read and discussed, either before the entire body or the special section to which the subject belonged: The Administration of Quinia in Pneumonia, by Dr. N. S. Davis; Extirpation of the Uterus, by Dr. Kimball, of Massachusetts; Treatment of some Diseases of the Female Urethra, by Dr. W. H. Byrd, of Illinois; Certain Operations for Vesico-Vaginal Fistulæ, by Dr. Bozeman; the Value of Extension in the Treatment of Fracture of the Femur, by Dr. Hodgen, of St. Louis; the Relations of Spiritualism to Medical Jurisprudence, by Dr. John P. Gray, of the Utica Asylum.

The paper which excited the most discussion seems to have been that read by Dr. Hodgen, on the treatment of the fractured femur. He pointed out the faults of plaster cases and pulley apparatuses before giving his own method of treatment. His conclusions were, that continued extension of the femur is essential; that this can not be secured by lateral supports of any kind, and that it can be by oblique suspension.

The evening of the first day was occupied by a number of receptions tendered the members of the association by the leading Chicago physicians.

### SECOND DAY'S PROCEEDINGS.

The revisions of the United States Pharmacopœia was the subject of Dr. E. R. Squibbs' address, on Wednesday morning. Our readers are already acquainted with his reasons for wishing this revision, so this part we shall not repeat. He made frequent reference to a pamphlet (Dr. Wood's) which appeared some months ago in opposition to the proposed revision. The paper, during its first half, treated of the decennial conventions and the manner in which they were called. Under the present arrangement, the convention is to meet in 1879, when a committee on revision will be appointed, who are to prepare an authoritative pharmacopœia, which no doubt, will be defended by all means legally available. The pamphlet pointed out that there would be two pharmacopœias, if the American Medical Association attempts to put one in the field. As to that point, Dr. Squibbs pointed out that Great Britain once had three pharmacopœias; and if there should come to be two in America, the doctrine of the "survival of the fittest" would, in its operation, take care of the best one. In May, 1880, a convention would meet in Washington which would be a convention, no matter how few its delegates, and it would have authority to revise the pharmacopœia.

Dr. P. G. Robinson, of Missouri, followed with a paper treating of the transmission of enteric fever by dairy milk. It instanced a case in which the animals in a dairy were watered at a stream which was polluted above by the excrements of a large number of workmen. The families using the milk were attacked. Dr. Robinson pointed out that great care should be taken in the milksupply of large cities, where chsldren were laid especially liable to injury from its impurities.

Dr. J. P. White, of Buffalo, New York, made a report from the section on obstetrics. It consisted for the greater part of a plea for the more frequent use of "that most humane of instruments, the obstetric forceps." He also exhibited an instrument, prepared by himself, which he claimed combined all the good points of all the other instruments in use, and none of the bad.

The section on practical medicine listened to and discussed an interesting report on clinical and meteorological records, by Dr. N. S. Davis and by Dr. Denison, on the influence of Colorado climate upon consumption. The latter paper is supplemental to the one he read in 1876. Dr. Denison is a warm advocate of Colorado air in the treatment of this affection, and presented many tables of statistics relative to the subject. He gave detailed accounts of twelve phthisical cases, of which half were favorable and half unfavorable to the Colorado climate. He had been led to the conclusion that consumption might be benefited by the Colorado atmosphere, not only in its early but under proper conditions, in its later stages. The influrnce of altitude on phthisis was favorable in the beginning of chronic inflammatory and hemorrhagic cases, and in cases allied to these. It was unfavorable as the disease was complicated by cardiac disease, associated with increased labor and abnormal activity.

The paper gave rise to a warm debate. One physician believed that patients who returned from Colorado were worse off than they would have been had they never gone. Another believed that those conditions which restored consumptives to health in Colorado were not climatic, and might be obtained at home. It was voted to refer the paper to the publishing committee, and request Dr. Denison to continue his studies, and embody his conclusions in a paper to be presented to the association next year.

Before the section on obstetrics, Dr. Bozeman submitted statistics relative to organic diseases as observed in Germany.

Dr. Marcy of Massachusetts, read an essay on "Congenital Absence and Imperfect Development of the Uterus," which was discussed by Drs. Webber, of Indiana; Staples, of Minnesota; Warner, Bozeman, Seymour, Sims, and Dean of New York, and Battey, of Georgia.

Drs. Cuttle, of Massachusetts, and Hildreth, of West Virginia, exhibited some new obstetrical instruments of their own device.

The section on surgery and anatomy met in Farwell Hall, the attendance at this section being greater than that at all the other sections combined. Dr. S. D. Gross read a paper prepared by his son, Dr. S. W. Gross, of Pennsylvania, on "Strictures of the Urethra from Masturbation, and its Pathological Significance." Dr. W. T. Briggs, of Tennessee, read a paper on "Medio-Bilateral Lithotomy." Dr. Lewis A. Sayre described his "Treatment of

Fractured Ribs by Extension and Expansion of the Thorax and Retention by Plaster of Paris Bandage."

Dr. Whipple, of New Jersey, described an operation in which he removed one and a half inches of the tibia and fibula of a patient. In the discussion of this case, Dr. Link, professor of anatomy in Indianapolis, described cases in his practice showing that the repair of a bone did not depend on the existance of periosteum.

The section on medical jurisprudence and psychology spent the entire afternoon in listening to and discussing a paper by Dr. R. J. Patterson, of Illinois, on the subject, ." Do Facts Justify the Recognition of Moral Insanity as a Distinct Form of Mental Disease ?"

Dr. Patterson's conclusions were adverse to such recognition. His paper contained statistics from a large number of insane asylums running back over several years, and showing a very marked diminution of so-called " moral insanity." Dr. Patterson objected to the recognition of moral insanity as a distinct form of mental disease, because there were no cases in which it was shown that a person suffered from "moral insanity," while the intellect was perfectly rational. No person would suffer by the denial of this recognition. The term "moral insanity" not only does no good, but it does positive harm by enabling unscrupulous lawyers to set up a specious plea in behalf of their clients, especially in cases of homicide, in which there is no question as to the soundness of the intellect. The cases of Sickles, McFarland, and many others were cited here. And finally very few of the highest authorities, medical or legal, recognized such a disease as moral insanity, and the plea of that disease is looked on with suspicion by experts.

In the discussion which followed, Drs. Gray, Thompson, Battey, and Knight agreed with the essayist while Drs. Seguin, of New York, and Buck, of Canada, contended for the existance of moral Insanity.

Dr. J. R. Black addressed the section of state medicine and public hygiene, on the Relation of Heredity to Race Degeneration and Improvement.

Dr. Comegys read a paper on state medicine, which particularly dwelt upon the necessity of rigid restrictions as regarded admission to practice. The State should exercise over all physicians a supervision so careful that no unqualified person could, by any possibility, foist himself upon the community.

### LAST DAY'S PROCEELINGS.

On Thursday morning the Association met with 650 delegates present.

The report of the committee on Necrology was read and referred.

The Treasurer, on retiring, reported unusually heavy expenses during the past year, but that he had a small surplus.

The Committee on Publication submitted a lengthy report, urging that a medically educated stenographer be employed to supply the copy of the proceedings for pamphlet publication, and making other recommendations.

The Committee on Prize Essays reported that only two had been submitted, and neither was worthy of a prize.

Dr. E. M. Hunt read a long paper on "State Medicine and Public Hygiene."

The Librarian reported that a number of new books had been added.

The qeestion of the revision of the Pharmacopœia was again discussed, but finally the whole subject was tabled.

The following officers were elected: President, Dr. T. G. Richardson, of Louisiana; Vice-Presidents, Drs. White, of New York, Gunn, of Illinois, Russell, of Connecticut, and Dunlap, of Ohio, with Chairmen and Secretaries of the various sections of the Convention.

The report of Drs. J. J. Woodward, U. S. A., and Edward Seguin, on the progress of uniformity in the means of observation and record, was submitted, as follows:

"During your last session you have voted that the question of uniformity in the means of medical observation and of medical records should be presented in your name to the International Medical Congress soon to meet in Philadelphia. This Congress received your communication with an interest enhanced by the warm recommendation of its illustrious President, Prof. Samuel D. Gross, in his inaugural address; and the International Medical Congress, in its turn, voted the sending of delegates to the next International Medical Congress, which is to meet in Geneva, September, 1877, with the special commission of pleading there the cause of medical uniformity. Such has been the offiicial progress of this question since your last meeting.

"Its technological progress consists (a) in the perfecting and cheapening of the sphygmograph, but not yet to the point of making it as popular as the thermometer. (b) In the reduction of urinometers and microscopes to a uniform standard and to pocket size. (c) In the invention of simple colorimeters and globulimeters, which separately realize the hopes induced fifteen years ago by the brilliant invention of Prof. Mantagazza. (d) The more important conquest of this year has been the acceptance of the metric system by the New York State Medical Society.

"It may not be out of place to add that the demand for instruments of positive observation has more than decupled in the last ten years, and that the use of uniform records of medical observation has increased from a few hundreds to thousands annually; a double progress entirely new to the initiative of this great progressive body, the American Medical Association.

"During the same period the pharmacists have made parallel efforts to bring uniformity in the products of their trade, which is an accessory to our art; and we must acknowledge that they are somewhat ahead of us. They will be strongly represented in the International Medical Congress of Geneva, and it would be more creditable for them than for us were they the first to agree upon the terms of this long longed-for uniformity on our own ground. Pharmacy would gain nothing and physic would lose much by leaving disconnected two movements which tend to establish uniformity in the pharmacopœia and in the practice of physic; both uniformities being twin sisters of the same spirit, the aspiration of the human mind towards the next synthesis.

"Therefore, your reporters on this subject propose that the American Medical Association send special delegates to the International Medical Congress at Geneva—as it did so effectively to the International Medical Congress of Brussels, in 1875,—to advocate the adoption of a progressive uniformity of means of medical observation and records, with the concurrence, if possible, of the members of this Congress who will be found there engaged in advocating the application of uniformity in this and other departments of science."

The Association adopted the conclusions of this report in its last general meeting, and fused the delegation to that effect with the other delegation to the permanent medical societies of Europe for 1877-'78, naming Dr. Edward Seguin, of New York, president of this mixed delegation.

The Association also adopted a resolution favoring the passage of the "Morrison Bill," now before Congress, repealing the tariff on quinine.

Buffalo was selected as the place of the next annual meeting, and the first Tuesday in June as the time.

The Association then adjourned, although the work of the sections was continued until late in the evening.

We have copied this condensed report from the *Cincinnati Clinic*, not being present to report or take notice of the proceedings.

